# Accuracy of diagnosis and health service codes in identifying frailty in Medicare data

**DOI:** 10.1186/s12877-020-01739-w

**Published:** 2020-09-07

**Authors:** Natalia Festa, Sandra M. Shi, Dae Hyun Kim

**Affiliations:** 1 Department of Internal Medicine, Massachusetts General Hospital, Boston, MA USA; 2 Division of Gerontology, Department of Medicine, Beth Israel Deaconess Medical Center, 1620 Tremont Street, Boston, MA 02120 USA; 3 Hinda and Arthur Marcus Institute for Aging Research, Hebrew SeniorLife, Boston, MA USA; 4 Division of Pharmacoepidemiology and Pharmacoeconomics, Department of Medicine, Brigham and Women’s Hospital, Boston, MA USA

**Keywords:** Frailty, Frailty phenotype, Older adult, Medicare administrative data

## Abstract

**Background:**

Capturing frailty within administrative claims data may help to identify high-risk patients and inform population health management strategies. Although it is common to ascertain frailty status utilizing claims-based surrogates (e.g. diagnosis and health service codes) selected according to clinical knowledge, the accuracy of this approach has not yet been examined. We evaluated the accuracy of claims-based surrogates against two clinical definitions of frailty.

**Methods:**

This cross-sectional study was conducted in a Health and Retirement Study subsample of 3097 participants, aged 65 years or older and with at least 12-months of continuous fee-for-service Medicare enrollment. We defined 18 previously utilized claims-based surrogates of frailty from Medicare data and evaluated each against clinical reference standards, ascertained from a direct examination: a deficit accumulation frailty index (FI) (range: 0–1) and frailty phenotype. We also compared the accuracy of the total count of 18 claims-based surrogates with that of a validated claims-based FI model, comprised of 93 claims-based variables.

**Results:**

19% of participants met clinical criteria for the clinical frailty phenotype. The mean clinical FI for our sample was 0.20 (standard deviation 0.13). *Hospital Beds and associated supplies* was the claims-based surrogate associated with the highest clinical FI (mean FI 0.49). Claims-based surrogates had low sensitivity ranging from 0.01 (*cachexia, adult failure to thrive, anorexia*) to 0.38 (*malaise and fatigue*) and high specificity ranging from 0.79 (*malaise and fatigue*) to 0.99 (*cachexia, adult failure to thrive, anorexia*) in discriminating the clinical frailty phenotype. Compared with a validated claims-based FI, the total count of claims-based surrogates demonstrated lower Spearman correlation with the clinical FI (0.41 [95% CI 0.38–0.44] versus 0.59 [95% CI, 0.56–0.61]) and poorer discrimination of the frailty phenotype (C-statistics 0.68 [95% CI, 0.66–0.70] versus 0.75 [95% CI, 0.73–0.77]).

**Conclusions:**

Claims-based surrogates, selected according to clinical knowledge, do not accurately capture frailty in Medicare claims data. A simple count of claims-based surrogates improves accuracy but remains inferior to a claims-based FI model.

## Background

Frailty is a major risk factor for adverse health outcomes among older adults, including falls, hospitalization, disability, institutionalization, and death [1–3]. The societal impact of frailty is projected to increase as its associated adverse outcomes accrue with population aging [4]. Due to its considerable individual and social costs, there is a critical need to reliably measure and monitor the epidemiologic burden of frailty [5]. As prevalent frailty increases with population aging, a standardized approach to its measurement is a precondition to informed health policy. Accurate recognition is similarly important to the primary and secondary prevention of this syndrome [6, 7]. In the absence of routine clinical assessment of frailty, administratively-derived measures that are well calibrated to clinical screening instruments will underpin its reliable identification and measurement.

Within administrative data, the identification of frail individuals is complicated by the absence of specific diagnosis codes to designate frailty [8]. This has resulted in the triangulation of frailty status according to the related but separate proxies of disability and comorbidity burden [1]. As such, there is increasing interest in measuring frailty through claims-based surrogates, including diagnosis, health-service, and procedural codes [9–11]. A series of claims-derived, model-based algorithms to identify frailty have been developed [12–19]. With comparable frequency, researchers have also attempted to identify frailty through the purposeful selection of administrative surrogates, as informed by clinical knowledge [16, 20–26]. For example, one such approach empirically segmented Medicare beneficiaries according to claims-based diagnoses that were inferred to represent frailty [25, 26]. This latter approach is often favored due to parsimony and ease of implementation [8, 25, 26].

Despite the accelerated development of instruments with which to capture frailty in administrative data, the performance of claims-based surrogates has not yet been evaluated against clinical frailty definitions. This study evaluates the accuracy of diagnosis and health service codes in identifying frailty, as defined by the frailty phenotype and a deficit-accumulation frailty index (FI) from a clinical examination, using Medicare data linked to the Health and Retirement Study (HRS). We then compare the performance of the selected administrative surrogates with that of a validated claims-based FI [12, 17, 27].

## Methods

### Study population

The HRS is a representative survey of community-dwelling adults in the United States, aged 50-years and older. The HRS longitudinally measures the health, cognitive, and functional status of its respondents [9–11]. All members of the HRS cohort are surveyed directly or by proxy on a biennial basis. All HRS subjects provide written informed consent prior to each interview, as well as oral consent in the presence of trained study professionals. The HRS is sponsored by the National Institute on Aging (grant NIA U01A6009740) and conducted by the University of Michigan with Institutional Review Board Approval.

This cross-sectional study includes 3097 community-dwelling HRS respondents who were aged ≥65-years, randomly selected to undergo clinical assessment of physical performance, and had 12 months of continuous fee-for-service Medicare enrollment prior to the 2008 HRS interview. In over 80% of participants, HRS data were linked to Medicare fee-for-service data. We used inpatient, outpatient, skilled nursing facility, home health, carrier, and durable medical equipment files that include International Classification of Diseases (ICD, version 9), Current Procedural Terminology (CPT), and Healthcare Common Procedure Coding System (HCPCS) codes collected in the year preceding the HRS interview. This study was approved by the Institutional Review Board at the Brigham and Women’s Hospital in Boston, Massachusetts.

### Clinical definitions of frailty

Our analysis utilized two clinical reference standards, the categorical frailty phenotype and the quantitative deficit-accumulation frailty index (FI). The measures for each reference standard were ascertained via direct clinical assessment of HRS participants by trained study professionals, as detailed within HRS survey data. The clinical frailty phenotype was defined according to the biologic syndrome model, with HRS-adapted criteria [28]. This model characterizes frailty according to the 5 domains of unintentional weight loss, exhaustion, low physical activity, slowness, and weakness (Supplementary Table S1) [29]. Individuals with impairments in ≥3 domains were considered frail, in 1–2 domains, pre-frail, and, in no domains, robust. We employed the standard deficit accumulation approach to calculate our quantitative clinical reference standard, the FI, utilizing 43 health deficits [30, 31]. The FI (range: 0–1) is the number of deficits present, divided by the total number of deficits measured (Supplementary Table S2). A higher FI score indicates a greater degree of frailty [27, 30, 31].

### Claims-based surrogates of frailty: diagnosis and health service codes

We selected 18 claims-based frailty surrogates that have commonly been applied in prior research (Table 1) [8, 25, 26]. These claims-based surrogates represent both clinical features and consequences of frailty, which closely map onto geriatric syndromes (*dementia, sensory disorders*) and functional deficits (*minor and severe ambulatory limitations, self-care impairment*) [19]. A surrogate measure was ascribed to a participant if he or she had been assigned a particular code within the 12 months preceding the HRS interview. These claims-based surrogates were examined individually and in a total count (“count approach”) (range 0–18) against clinical definitions of frailty.

**Table 1:** Claims-Based Surrogates of Frailty

Conditions Suggestive of Frailty	ICD-9/10 or HCPCS Codes
Pressure ulcer	(ICD9) 707.0X, 707.2X, (ICD10) L89.XX
Cachexia	(ICD9) 799.4, (ICD10) R64
Adult failure to thrive	(ICD9) 783.7, (ICD10) R62.7
Muscle weakness	(ICD9) 728.87, (ICD10) M62.81
Debility	(ICD9) 799.3, (ICD10) R54
Difficulty in walking	(ICD9) 719.7, (ICD10) R26.2
History of fall	(ICD9) V15.88, (ICD10) Z91.81
Abnormality of gait	(ICD9) 781.2, (ICD10) R26.0, R26.1, R26.89, R26.9
Anorexia	(ICD9) 783.0, (ICD10) R63.0
Abnormal loss of weight and underweight	(ICD9) 783.21, 783.22 (ICD10) R63.4, R63.6
Muscular wasting and disuse atrophy	(ICD9) 728.2, (ICD10) M62.50
Senility without mention of psychosis	(ICD9) 797, (ICD10) R41.81
Malaise and fatigue	(ICD9) 780.79, (ICD10) R53.1, R53.81, R53.83
Hospital beds and associated supplies	(HCPCS) E0250-E0373
Wheelchairs, components, and accessories	(HCPCS) E0950-E1298, E2201-E2294, E2300-E2399, E2601-E2621, K0001-K0195, K0669
Accessories for oxygen delivery devices	(HCPCS) E1353-E1406
Walking aids and attachments	(HCPCS) E0100-E0159
Transportation services including ambulance	(HCPCS) A0021-A0999

*Abbreviations*: *HCPCS* Healthcare Common Procedure Coding System, *ICD* International Classification of Diseases

### Claims-based frailty index: a model-based approach

We estimated a previously validated claims-based FI [12, 17, 27], which utilizes ICD, CPT, and HCPCS codes in Medicare claims data over a 12-month period. The selected claims-based FI has previously outperformed alternative claims-based FIs in its agreement with the clinical frailty phenotype and clinical FI [27]. The weights assigned to 93 claims-based variables in the model were estimated from a lasso penalized regression model. A SAS program to calculate this claims-based FI from the Medicare data or United States commercial insurance data is available (https://dataverse.harvard.edu/dataset.xhtml?persistentId=doi:10.7910/DVN/HM8DOI). We compared the performance of the claims-based FI model to that of the total count of claims-based surrogates in predicting clinical frailty definitions.

### Statistical analysis

Analyses were performed using R version 3.6.0 and Stata version 14. We summarized population characteristics in mean and standard deviation (SD) or proportions. Because the frailty phenotype was undetermined for 827 individuals with missing measures of gait speed and grip strength, we performed a single multivariable imputation (Stata mi impute chained command) to assign the missing frailty phenotype with available information (demographic characteristics, chronic conditions, and self-reported health and functional status).

We estimated the proportion of individuals with each claims-based surrogate of frailty. For each claims-based surrogate, we calculated the associated mean and standard deviation (SD) of the clinical FI. We then determined sensitivity, specificity, positive predictive value (PPV), and negative predictive value (NPV) for the clinical frailty phenotype. We next examined the distribution of the total count of claims-based surrogates of frailty, the “count approach.” For each threshold of the count approach (i.e., an individual is considered frail if the individual has claims-based surrogates of frailty equal to or more than this threshold), we calculated the mean and SD of the clinical FI, as well as the sensitivity, specificity, PPV, and NPV for the clinical frailty phenotype. As a comparison, we examined the performance of the claims-based FI model (“model-based approach”) at various cutpoints. An optimal cutpoint was defined as the threshold that achieved the highest combined sensitivity and specificity. In addition, we compared the count approach and the model-based approach by estimating Spearman correlation with the clinical FI and C-statistics for the clinical frailty phenotype. We determined the 95% confidence intervals (CIs) of Spearman correlation and C-statistics through 1000 bootstrap resampling.

## Results

### Characteristics of study population

The 3097 HRS respondents included in the analysis had a mean age of 75.7 years (SD 7.2). Women and Caucasian respondents comprised 58.1% (*n* = 1798) and 86.6% (*n* = 2683) of the sample, respectively. The mean FI for the overall sample was 0.20 (SD 0.13). Among 2270 respondents with complete data on the components of frailty phenotype, the proportions of robust, pre-frail, and frail individuals were 21.9% (*n* = 679), 40.4% (*n* = 1252), and 10.9% (*n* = 339). After imputation, the corresponding proportions were 27.6% (*n* = 856), 53.4% (*n* = 1654), and 19.0% (*n* = 587), suggesting that those with missing data had more severe frailty.

### Claims-based surrogates of frailty

The most prevalent claims-based surrogates were *malaise and fatigue* (23.9%), *transportation services including ambulance* (9.7%), *abnormality of gait* (7.5%), *muscle weakness* (5.3%), and *accessories for oxygen use devices* (4.8%) (Table 2). Claims-based surrogates associated with the highest mean clinical FI were *hospital beds and associated supplies* (FI 0.49), *pressure ulcer* (FI 0.43), *cachexia* (FI 0.43), *wheelchairs, components, and accessories* (FI 0.41)*,* and *adult failure to thrive* (FI 0.36). Claims-based surrogates were specific but not sensitive in identifying the clinical frailty phenotype. Except for *malaise and fatigue* (specificity 0.79), all surrogates had specificity greater than 0.90. Surrogates with the highest sensitivity were *malaise and fatigue* (sensitivity 0.38) and *transportation services including ambulance* (sensitivity 0.24). At the imputed prevalence of phenotypic frailty (19%) within our sample, the highest PPV was observed for *cachexia* (PPV 0.75), *hospital beds and associated supplies* (PPV 0.73), and *wheelchairs, components, and accessories* (PPV 0.63). The NPV was greater than 0.80 for all surrogates.

**Table 2:** Performance of Claims-Based Surrogates of Frailty Against Clinical Frailty Assessment in the Health and Retirement Study-Medicare Data

Claims-Based Surrogates of Frailty^**a**^	Sample Size (%)	Clinical FI Mean (SD)	Clinical Frailty Phenotype			
			Sensitivity	Specificity	PPV	NPV
Hospital beds and associated supplies	44 (1.4%)	0.49 (0.14)	0.06	0.99	0.73	0.82
Pressure ulcer	39 (1.3%)	0.43 (0.17)	0.04	0.99	0.59	0.82
Cachexia	< 10 (< 0.3%)	0.43 (0.11)	0.01	> 0.99	0.75	0.81
Wheelchairs, components, and accessories	63 (2.6%)	0.41 (0.15)	0.09	0.99	0.63	0.82
Adult failure to thrive	< 10 (< 0.3%)	0.36 (0.17)	0.01	> 0.99	0.57	0.81
Muscle weakness	164 (5.3%)	0.34 (0.16)	0.12	0.96	0.44	0.82
Accessories for oxygen delivery devices	149 (4.8%)	0.34 (0.16)	0.11	0.97	0.44	0.82
Debility	50 (1.6%)	0.34 (0.15)	0.04	0.99	0.48	0.81
Difficulty in walking	135 (4.4%)	0.32 (0.15)	0.10	0.97	0.44	0.82
Walking aids and attachments	130 (4.2%)	0.32 (0.14)	0.10	0.97	0.47	0.82
History of fall	56 (1.8%)	0.31 (0.16)	0.04	0.99	0.41	0.81
Transportation services including ambulance	300 (9.7%)	0.31 (0.16)	0.24	0.93	0.46	0.84
Abnormality of gait	231 (7.5%)	0.31 (0.15)	0.18	0.95	0.45	0.83
Anorexia	15 (0.5%)	0.30 (0.22)	0.01	> 0.99	0.33	0.81
Abnormal loss of weight and underweight	112 (3.6%)	0.28 (0.16)	0.08	0.97	0.44	0.82
Muscular wasting and disuse atrophy	23 (0.7%)	0.28 (0.12)	0.02	0.99	0.39	0.81
Senility without mention of psychosis	< 10 (< 0.3%)	0.26 (0.21)	< 0.01	> 0.99	0.33	0.81
Malaise and fatigue	740 (23.9%)	0.25 (0.15)	0.38	0.79	0.30	0.84

*Abbreviations*: *FI* frailty index, *HCPCS* Healthcare Common Procedure Coding System, *ICD* International Classification of Diseases, *NPV* negative predictive value, *PPV* positive predictive value, *SD* standard deviation

^a^Claims-based surrogates of frailty were defined as occurrence of the respective codes in a 12-month period in any of the inpatient, outpatient, skilled nursing facility, home health, carrier, and durable medical equipment datasets

### Total count of claims-based surrogates of frailty

**Fig. 1:**
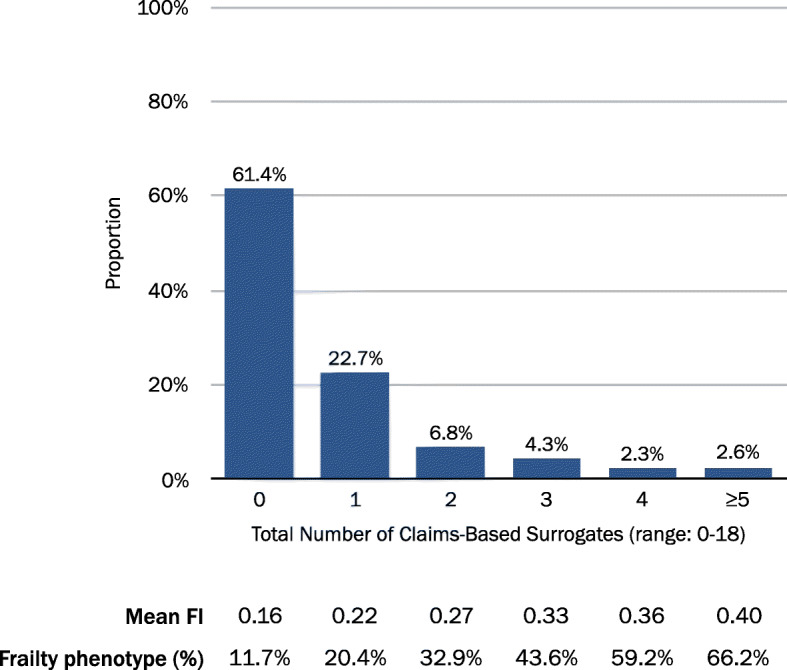
Classification of Frailty by Total Count of Claims-Based Frailty Surrogates in the Health and Retirement Study-Medicare Data. Abbreviations: FI, frailty index

The proportions of respondents whose administrative data indicated a total count of 0, 1, 2, 3, 4, and ≥ 5 claims-based frailty surrogates were 61.4, 22.7, 6.8, 4.3, 2.3, 2.6%, respectively (Fig. 1). The corresponding mean clinical FI ranged from 0.16 (0 surrogates) to 0.40 (≥5 surrogates). The associated proportion of the clinical frailty phenotype ranged from 11.7% (0 surrogates) to 66.2% (≥5 surrogates).

An increasing count of claims-based frailty surrogates corresponded to a decrease in sensitivity, from 0.62 (≥1 surrogates) to 0.09 (≥5 surrogates), and an increase in specificity, from 0.67 (≥1 surrogates) to 0.99 (≥5 surrogates), in discriminating the clinical frailty phenotype (Table 3). An optimal cutpoint of ≥1 surrogate achieved the highest combined sensitivity (0.62) and specificity (0.67) for the count approach. At the prevalence of the frailty phenotype (19.0%) in the study population, the total count of claims-based surrogates showed a modest PPV of 0.31 (cutpoint ≥1) to 0.66 (cutpoint ≥5). The NPV was greatest for a threshold of ≥1 surrogate (0.88).

**Table 3:** Performance of Total Count of Claims-Based Surrogates of Frailty versus a Model-Based Claims-Based Frailty Index Against Clinical Frailty Assessment in the Health and Retirement Study-Medicare Data

Threshold to Define Frailty	Positive for Frailty N (%)	Clinical FI Mean (SD)	Clinical Frailty Phenotype			
			Sensitivity	Specificity	PPV	NPV
**A. Total count of claims-based surrogates of frailty (range: 0–18)**^**a**^						
≥ 1	1196 (38.6)	0.26 (0.15)	0.62	0.67	0.31	0.88
≥ 2	494 (16.0)	0.32 (0.16)	0.38	0.89	0.45	0.86
≥ 3	284 (9.2)	0.36 (0.15)	0.26	0.95	0.54	0.85
≥ 4	151 (4.9)	0.38 (0.15)	0.16	0.98	0.63	0.83
≥ 5	80 (2.6)	0.40 (0.16)	0.09	0.99	0.66	0.82
**B. Model-based claims-based frailty index (range: 0–1)**^**b**^						
≥ 0.15	1464 (47.3)	0.26 (0.14)	0.76	0.59	0.30	0.91
≥ 0.20	632 (20.4)	0.33 (0.15)	0.47	0.86	0.44	0.87
≥ 0.25	302 (9.8)	0.37 (0.15)	0.30	0.95	0.58	0.85
≥ 0.30	133 (4.3)	0.44 (0.15)	0.16	0.99	0.72	0.83
≥ 0.35	70 (2.3)	0.46 (0.16)	0.09	0.99	0.76	0.82

*Abbreviations*: *FI* frailty index, *NPV* negative predictive value, *PPV* positive predictive value, *SD* standard deviation

^a^The optimal cutpoint of the count approach for frailty phenotype was ≥1, which achieved a sensitivity of 0.62 and a specificity of 0.67

^b^The optimal cutpoint of CFI for frailty phenotype was ≥0.17, which achieved a sensitivity of 0.66 and a specificity of 0.72

### Comparison with a model-based approach

**Fig. 2:**
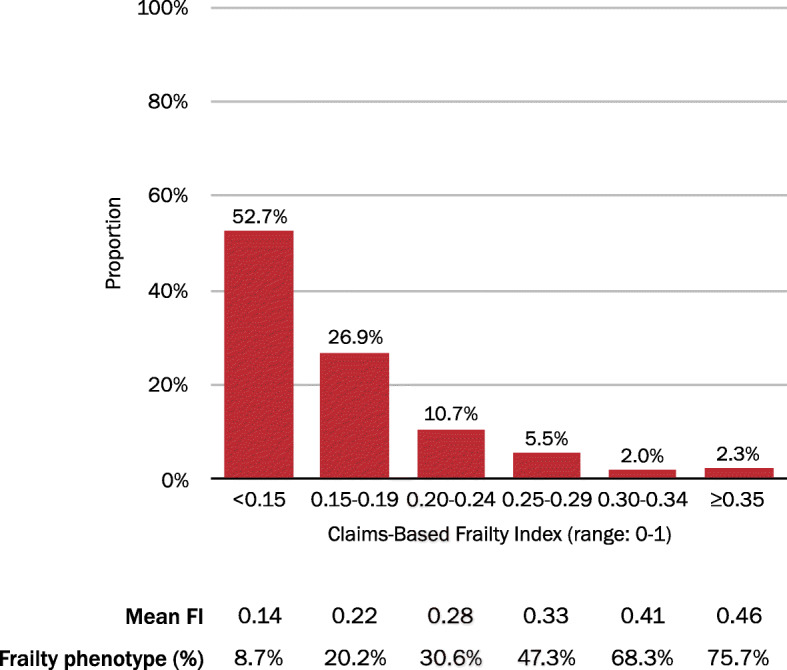
Classification of Frailty by a Claims-Based Frailty Index Model in the Health and Retirement Study-Medicare Data. Abbreviations: FI, frailty index

The proportions of respondents with claims-based FI scores < 0.15, 0.15–0.19, 0.20–0.24, 0.25–0.29, 0.30–0.34, and ≥ 0.35 were 52.7, 26.9, 10.7, 5.5, 2.0, and 2.3%, respectively (Fig. 2). The associated mean clinical FI ranged from 0.14 for those with a claims-based FI score < 0.15 to 0.46 for those with a claims-based FI score ≥ 0.35. The proportion of subjects meeting clinical criteria for the frailty phenotype increased in accordance with claims-based FI scores, ranging from 8.7% for those with a claims-based FI score < 0.15 to 75.7% those with a claims-based FI score ≥ 0.35.

**Fig. 3:**
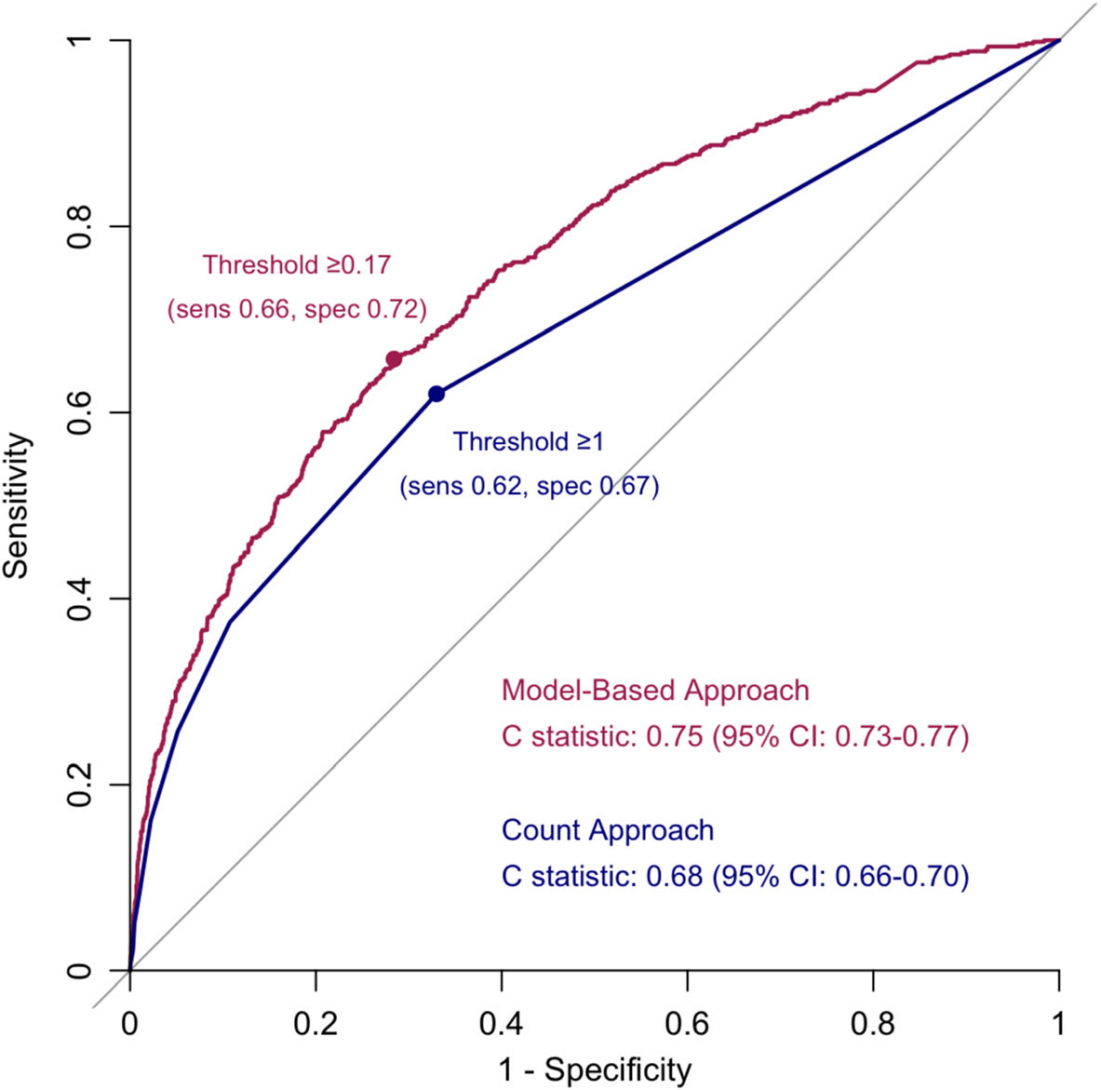
Receiver Operating Characteristic Curves of Total Count of Claims-Based Frailty Surrogates (Count Approach) versus a Claims-Based Frailty Index Model (Model-Based Approach) in Identifying the Frailty Phenotype. Abbreviations: CI, confidence interval; sens, sensitivity; spec, specificity. Legend: The count approach demonstrated a lower C-statistic than the model-based approach in identifying the frailty phenotype. The optimal cutpoint of the count approach was ≥1, which achieved a sensitivity of 0.62 and a specificity of 0.67. The optimal cutpoint of model-based approach was ≥0.17, which achieved a sensitivity of 0.66 and a specificity of 0.72

The Spearman correlation with the clinical FI was lower for the count approach (correlation coefficient 0.41; 95% CI 0.38–0.44) than for the claims-based FI model (correlation coefficient 0.59; 95% CI 0.56–0.61). The count approach demonstrated poorer discrimination (C-statistic 0.68; 95% CI 0.66–0.70) of the clinical frailty phenotype than did the claims-based FI model (C-statistic 0.75; 95% CI 0.73–0.77) (Fig. 3). An optimal cutpoint for the claims-based FI model of ≥0.17 achieved the highest combined sensitivity (0.66) and specificity (0.72) in discriminating the clinical frailty phenotype.

## Discussion

Although certain claims-based surrogates (*hospital beds and associated supplies*; *pressure ulcer*; *cachexia; wheelchairs, components, and accessories*; and *adult failure to thrive*) appropriately identified Medicare beneficiaries with a moderate-to-severe clinical FI (mean FI > 0.35), only a minority (< 3%) of the population had been assigned those codes. Similarly, these surrogates demonstrated low sensitivity (0.01–0.38) and high specificity (0.80–1.00) in discerning the clinical frailty phenotype. Our findings suggest that claims-based surrogates that have been selected according to clinical expertise are suboptimal in identifying frail older adults within Medicare data. While the novel count approach improved sensitivity and specificity for the frailty phenotype, it underperformed a claims-based FI model.

Our selected claims-based surrogates of frailty represent either clinical manifestations or consequences of frailty that are recognizable by clinicians and, as such, have been commonly utilized in prior research. The most common claims-based surrogate of frailty, *malaise and fatigue* (23.9% of respondents), was the most sensitive (sensitivity 0.38) indicator of the frailty phenotype. The most specific indicators (specificity ≥0.99) of the frailty phenotype *(hospital beds and associated supplies; pressure ulcer; cachexia; wheelchairs, components, and accessories; adult failure to thrive*) were expectedly associated with a greater mean clinical FI. The low sensitivity of these codes may reflect the lack of routine frailty screening in clinical practice or variability in the documentation of its clinical features in administrative processes [32]. Therefore, without the widespread clinical adoption and documentation of standardized frailty screening, individual claims-based surrogates are likely to remain an insufficient and inaccurate indicator of frailty status.

Despite their poor sensitivity, there was parity between the studied administrative measures and the underpinning theory of phenotypic frailty. The most common (23.9% of respondents) and sensitive (0.35) claims-based surrogate, *Malaise and fatigue*, is consistent with several definitional criteria of phenotypic frailty, including *weakness* and *exhaustion.* The most specific predictors of the frailty phenotype *(Hospital beds and associated supplies; Pressure ulcer; Wheelchairs, components, and accessories; Cachexia; Adult failure to thrive*) maintained similar parity with the phenotypic criteria of *a low level of activity, slowness,* and *weakness*. The most specific indicators of frailty also aligned with the phenotypic criterion of *weight-loss*, namely, *Cachexia* and *Adult failure to thrive*. Each of the most specific indicators similarly corresponded to domains within the esteemed and proprietary JEN-Frailty Index, such as *minor ambulatory limitations, severe ambulatory limitations,* and *self-care impairment* [19].

The results of our study are informative to health services researchers and health care systems that aim to better characterize utilization and outcomes among high-need older adults through the reliable identification of frail individuals. Various efforts to identify frailty through the use of claims-based surrogates have preceded our analysis [16, 20–26]. Our results suggest that the novel count approach identifies frailty with greater accuracy than does the clinically-informed selection of claims-based surrogates. Despite its appealing parsimony and preferable performance to individual claims-based surrogates, the count approach underperformed a claims-based FI model. Considering the superior performance of the claims-based FI model, future research is needed to distinguish among recently developed and validated models [12–18]. Available claims-based FI model differ considerably with respect to their chosen reference standard (i.e. disability, frailty phenotype, or deficit-accumulation FI), data utilized for estimation and validation, and performance characteristics [27].

Our study has a few limitations. First, we assessed the performance of ICD-9 claims-based surrogates of frailty against clinical frailty definitions. Performance may be affected by associated changes in coding practice. For the purpose of future research, ICD-10 diagnoses corresponding to our analysis are summarized (Table 1). Second, we imputed the frailty phenotype for 26.7% of the study population due to missing measurements of gait speed or grip strength. The analysis of imputed data assumes that the missingness is not related to the frailty level after accounting for all measured variables, however, this missing-at-random assumption may not be satisfied. Nonetheless, exclusion of those with missing frailty data from our analysis would have resulted in a biased estimation of the performance of claims-based surrogates. Third, our study population was limited to Medicare fee-for-service beneficiaries within the HRS sample. Therefore, generalizability to beneficiaries of Medicare Advantage or a commercial insurance plan remains unclear.

## Conclusions

Our study advances knowledge of the performance characteristics of claims-based surrogates in discriminating frailty status. Our results suggest that claims-based frailty surrogates offer poor sensitivity, rendering these poor screening measures at the population level. Employing a total count of claims-based surrogates is an efficient method that improves sensitivity, while moderately compromising specificity. Despite its attractive parsimony and simplicity, the discriminatory power of the count approach remains inferior to that of the claims-based FI model. Our results suggest that the identification of frailty through the claims-based FI model, which assigns appropriate weights to a larger assemblage of variables, should supplant the selection of surrogates on the sole basis of clinical knowledge.

## Supplementary information

**Additional file 1: Table S1.** Clinical Reference Standard: Frailty Phenotype. **Table S2.** Clinical Reference Standard: Deficit Accumulation Frailty Index.

## Data Availability

The Health and Retirement Study data analyzed during this study are available from the University of Michigan, pending Institutional Review Board approval.

## Abbreviations

HRS: Health and Retirement Study
FI: Frailty Index
SD: Standard Deviation
PPV: Positive Predictive Value
NPV: Negative Predictive Value
