# Supplementary material for: Accuracy of diagnosis and health service codes in identifying frailty in Medicare data
Source: BMC Geriatr. 2020 Sep 7;20:329. doi: 10.1186/s12877-020-01739-w (PMC7487915; doi:10.1186/s12877-020-01739-w)
Supplement: Supplementary file 1 — Additional file 1: Table S1. Clinical Reference Standard: Frailty Phenotype. Table S2. Clinical Reference Standard: Deficit Accumulation Frailty Index. [file 12877_2020_1739_MOESM1_ESM.docx]

**Supplementary Table S1. Clinical Reference Standard: Frailty Phenotype**

| **Frailty Phenotype ^a^** |
| --- |
| Unintentional weight loss |
| Exhaustion |
| Low physical activity |
| Slowness |
| Weakness |

a Data from direct clinical frailty assessment for our selected subjects within the Health and Retirement Study was utilized to determine phenotypic frailty, according to the standard Fried definition.^28^

**Supplementary Table S2. Clinical Reference Standard: Deficit Accumulation Frailty Index**

| **Evaluated Deficits ^b^** | **Scoring** |
| --- | --- |
| **Medical history** |  |
| Angina or chest pains due to your heart | 1 (yes), 0 (no) |
| Arthritis or rheumatism | 1 (yes), 0 (no) |
| Cancer or malignant tumor, excluding minor skin cancer | 1 (yes), 0 (no) |
| Chronic kidney disease based on cystatin C-based GFR <60 | 1 (yes), 0 (no) |
| Chronic lung disease (chronic bronchitis or emphysema) | 1 (yes), 0 (no) |
| Congestive heart failure | 1 (yes), 0 (no) |
| Depression based on the 8-item Center for Epidemiological Studies-Depression scale >4 or use of tranquilizers, antidepressants, or pills for nerves | 1 (yes), 0 (no) |
| Diabetes or high blood sugar | 1 (yes), 0 (no) |
| Myocardial infarction or heart attack | 1 (yes), 0 (no) |
| Fall | 1 (yes), 0 (no) |
| Hearing rated as poor | 1 (yes), 0 (no) |
| Hip fracture | 1 (yes), 0 (no) |
| Hypertension or high blood pressure | 1 (yes), 0 (no) |
| Memory-related disease | 1 (yes), 0 (no) |
| Psychiatric problems | 1 (yes), 0 (no) |
| Stroke | 1 (yes), 0 (no) |
| Urinary incontinence | 1 (yes), 0 (no) |
| Vision rated as poor or legally blind | 1 (yes), 0 (no) |
| Polypharmacy based on use of ≥5 prescription drugs | 1 (yes), 0 (no) |
| **Functional status** |  |
| Difficulty with dressing | 1 (yes), 0 (no) |
| Difficulty with bathing | 1 (yes), 0 (no) |
| Difficulty with walking | 1 (yes), 0 (no) |
| Difficulty with eating | 1 (yes), 0 (no) |
| Difficulty with transferring | 1 (yes), 0 (no) |
| Difficulty with toileting | 1 (yes), 0 (no) |
| Difficulty with meal preparation | 1 (yes), 0 (no) |
| Difficulty with shopping | 1 (yes), 0 (no) |
| Difficulty with telephone | 1 (yes), 0 (no) |
| Difficulty with medication management | 1 (yes), 0 (no) |
| Difficulty with housework | 1 (yes), 0 (no) |
| Difficulty with financial management | 1 (yes), 0 (no) |
| Difficulty with walking several blocks | 1 (yes), 0 (no) |
| Difficulty with lifting 10 lbs | 1 (yes), 0 (no) |
| Difficulty with climbing up 10 steps | 1 (yes), 0 (no) |
| Difficulty with kneeling, stooping, or crouching | 1 (yes), 0 (no) |
| Difficulty with reaching arms above shoulder | 1 (yes), 0 (no) |
| Difficulty with gripping small objects | 1 (yes), 0 (no) |
| Difficulty with pushing large objects | 1 (yes), 0 (no) |
| **Performance tests** |  |
| Telephone interview of cognitive status examination (24 points based on orientation, backward counting, immediate recall, serial 7 subtraction, and naming) | 1 (0-12 points)  0.7 (13-15 points)  0.3 (16-19 points)  0 (20-24 points) |
| Usual gait speed from 8-foot walk | 1 (<0.60 m/s)  0.5 (0.60-0.79 m/s)  0 (≥0.80 m/s) |
| Dominant handgrip strength | Male:  1 (<26.0 kg)  0.5 (26.0-31.9 kg)  0 (≥32.0 kg)  Female:  1 (<16.0 kg)  0.5 (16.0-20.0 kg)  0 (≥20.0 kg) |
| **Nutritional status** |  |
| Weight loss ≥10% over 2 years | 1 (yes), 0 (no) |
| Body mass index < 21 kg/m^2^ | 1 (yes), 0 (no) |

b Data from direct clinical frailty assessment for our selected subjects within the Health and Retirement Study was utilized to calculate a quantitative deficit accumulation frailty index for each subject.^27, 30, 31^
